# Differential Effects of Commercial Food-Grade Carrageenan Preparations on DSS-Induced Colitis and Gut Microbiota in Mice

**DOI:** 10.3390/foods15071131

**Published:** 2026-03-25

**Authors:** Jiyu Nie, Danying Chen, Chengfeng Yu, Zengliang Jiang, Haibo Pan, Xingqian Ye, Shiguo Chen

**Affiliations:** 1 National-Local Joint Engineering Laboratory of Intelligent Food Technology and Equipment, Zhejiang Key Laboratory for Agro-Food Processing, Zhejiang Engineering Laboratory of Food Technology and Equipment, College of Biosystems Engineering and Food Science, Zhejiang University, Hangzhou 310058, China; 2 Innovation Center of Yangtze River Delta, Zhejiang University, Jiaxing 314102, China; 3 Zhejiang University Zhongyuan Institute, Zhengzhou 450000, China

**Keywords:** carrageenan preparations, colitis, gut microbiota, short-chain fatty acids

## Abstract

Carrageenan (CGN) is widely used in processed foods and is typically supplied as a commercial preparation blended with other hydrocolloids to improve gelling properties, rather than as a single purified polymer. However, safety evaluations and mechanistic studies have largely focused on CGN in isolation; as a result, the biological effects of commercial CGN preparations (CGNPs) under realistic exposure conditions are still insufficiently characterized. In this study, the structural characteristics of three commercial food-grade CGNPs intended for meat products, soft sweets, and jelly were investigated. Furthermore, their effects on colitis were assessed, along with their impacts on the gut microbiota and related metabolites. The results indicated that all three CGNPs were κ-type, but differed in monosaccharide composition and molecular weight, which may contribute to their biological differences. In vivo, the CGNP intended for soft sweets significantly reduced the disease activity index (*n* = 6/group, *p* < 0.05) and helped maintain colon length (*n* = 6/group, *p* < 0.05). This CGNP also markedly reduced the abundance of *Escherichia-Shigella* and *Helicobacter*, while increasing propionate levels (*n* = 6/group, *p* < 0.05). In contrast, CGNPs intended for meat products and jelly tended to exacerbate colitis and increased the abundance of *Enterococcus*, a genus associated with colitis. These findings reveal the application-specific biological effects of commercial food-grade CGNPs and provide a basis for optimizing the application of these preparations in the food industry.

## 1. Introduction

Carrageenan (CGN), a natural linear sulfated polysaccharide extracted from red seaweed, is widely used in the food industry [1]. Its backbone consists of alternating β-D-galactose (G unit) and α-D-galactose (D unit) residues, and in some types, the D unit can further cyclize to form 3,6-anhydro-galactose (DA). The most common CGN types include κ, ι, and λ. In κ-CGN, the repeating disaccharide unit is typically represented as G4S-DA, consisting of β-D-galactose-4-sulfate (G4S) alternating with α-D-3,6-anhydro-galactose (DA). ι-CGN has a structure similar to that of κ-CGN, except that the DA unit is further sulfated at the C-2 position, forming G4S-DA2S (α-D-3,6-anhydro-galactose-2-sulfate). In contrast, λ-CGN contains three sulfate ester groups per repeating disaccharide unit and lacks DA. Its commonly described structure is G2S-D2S6S, in which the G unit is sulfated at the C-2 position (G2S), whereas the D unit is disulfated at the C-2 and C-6 positions (D2S6S) [1]. The structural diversity of CGN is primarily determined by the number and position of sulfate ester groups in the repeating disaccharide units and the presence or absence of the DA unit, which directly influence its solubility and gelation properties [1]. Numerous studies have demonstrated that long-term intake of CGN may induce colitis in experimental animals [2,3]. Notably, most of the existing evidence has been generated using CGN in isolation [4,5,6]. However, in commercial food applications, CGN is usually mixed with other hydrocolloids [7].

Blending CGN with other hydrocolloids is a common formulation strategy in commercial foods to adjust gel strength, water-holding capacity, and storage stability. For instance, CGN was blended with locust bean gum (LBG), konjac gum (KG), or xanthan gum (XG) to produce gels with improved strength and water retention capacity [8]. This formulation strategy is widely employed in the preparation of Asian dessert jellies [9]. In addition, semi-refined CGN (SRC) contains more cellulose than refined CGN and is primarily used in processed meat products to enhance product value [9]. In ice cream products, CGN is often combined with carboxymethyl cellulose (CMC), guar gum (GG), sodium alginate (SA), and XG to improve texture and storage stability [10], whereas in yogurt products, it serves as a stabilizer or thickening agent, occasionally combined with LBG and gum arabic (GA) [11]. Since commercial CGNPs used in the industry consist of multiple hydrocolloids, it is crucial to evaluate their biological effects under realistic exposure conditions.

Beyond their roles in texture design, hydrocolloid combinations can also reshape gut microbial fermentation and the resulting metabolic outputs. For example, the addition of XG has been reported to modify the fermentation characteristics of GG, thereby promoting the proliferation of beneficial bacteria [12]. This is particularly relevant in the context of colitis, in which gut microbiota dysbiosis is a key feature and is often accompanied by reduced microbial diversity, altered community structure, and disrupted microbial metabolite production [13,14]. Among these metabolites, short-chain fatty acids (SCFAs) are important for maintaining intestinal barrier integrity and immune homeostasis. Therefore, we aimed to investigate the effects of commercial food-grade carrageenan preparations (CGNPs) that may contain other hydrocolloids on colitis, gut microbiota, and SCFA production, as this aspect remains insufficiently studied [15,16,17,18,19].

We hypothesized that structural differences among commercial CGNPs would lead to distinct impacts on inflammation, microbiota, and SCFA production. Therefore, we analyzed the structural characteristics of CGNPs intended for meat products, soft sweets, and jelly and investigated their effects on gut microbiota composition and SCFAs profiles. These findings aim to provide new theoretical insights into the safety assessment of CGN in food applications and may inform dietary management strategies for IBD.

## 2. Materials and Methods

### 2.1. Materials and Reagents

CGNPs were purchased from Fujian (Brand A), Shandong (Brand B), Guangdong (Brand C), and Henan (Brand D) Provinces, China. The monosaccharide standards, including arabinose (Ara), fucose (Fuc), galactose (Gal), rhamnose (Rha), glucose (Glc), mannose (Man), and xylose (Xyl), were purchased from Sigma-Aldrich (Shanghai, China). Dextran sulfate sodium (DSS; 36,000–50,000 Da) was purchased from MP Bio (Santa Ana, CA, USA). The SCFA standards, including acetate, butyrate, propionate, and valerate, were purchased from Aladdin (Shanghai, China). All other reagents used in this study were of analytical grade and were purchased from Aladdin (Shanghai, China).

### 2.2. Structural Characterization of CGNPs

#### 2.2.1. FTIR Analysis

The FT-IR spectra of all samples were determined using a Nicolet iS50 FT-IR spectrometer (Thermo Fisher Scientific, Waltham, MA, USA). Briefly, the CGNP samples were placed on the infrared-transmitting crystal surface of the attenuated total reflectance (ATR) attachment and scanned in the range of 400–4000 cm^−1^.

#### 2.2.2. Monosaccharide Composition Analysis

The samples were prepared with 4 M trifluoroacetic acid at 110 °C for 8 h and subjected to monosaccharide composition analysis by high-performance anion-exchange chromatography with pulsed amperometry detection (HPAEC-PAD) [20]. A Dionex CarboPac PA-10 column (250 mm × 4 mm, Thermo Fisher Scientific, Waltham, MA, USA) with a PA-10 guard column (Thermo Fisher Scientific, Waltham, MA, USA) was used.

The mobile phase consisted of (A) ultrapure water, (C) 200 mmol/L sodium hydroxide, and (D) 24 mmol/L sodium hydroxide containing 100 mmol/L sodium acetate. The elution conditions were as follows: 13% C 15 min; 15–35 min, 100% D. The flow rate was 1 mL/min, the injection volume was 25 μL, and the temperature was 35 °C.

#### 2.2.3. Molecular Weight Analysis

The molecular weight was determined by high-performance size exclusion chromatography (SEC) equipped with multi-angle laser light scattering (DAWN HELEOS II, Wyatt Technology, Santa Barbara, CA, USA) and a refractive index (RI) detector [21]. The system was equipped with a Shodex OHpak SB-G 6B guard column (Shodex, Tokyo, Japan), an SB-804 HQ column (10 μm, 8.0 × 300 mm, Shodex, Tokyo, Japan), and an SB-806 HQ column (13 μm, 8.0 × 300 mm, Shodex, Tokyo, Japan). The mobile phase was 0.15 mol/L NaCl. The flow rate was 0.5 mL/min, the injection volume was 50 μL, and the temperature was 40 °C. Samples were prepared at 3 mg/mL in the mobile phase and analyzed for 60 min.

#### 2.2.4. NMR Spectroscopy

NMR analysis was performed using an Agilent DD2-600 spectrometer (Agilent Technologies, Santa Clara, CA, USA) at 500 MHz and 333.15 K. Briefly, 10 mg of the sample was dissolved in 0.6 mL of D_2_O and subsequently freeze-dried. This process was repeated twice. Finally, the sample was dissolved in 0.6 mL of D_2_O and transferred into an NMR tube for analysis.

### 2.3. Animal Experimental Design

All procedures for animal use were conducted in strict compliance with the National Research Council’s Guide for the Care and Use of Laboratory Animals and approved by the Institutional Animal Care and Use Committee of Zhejiang Province (Approval No.: ZJCLA-IACUC-20010970). Male C57BL/6J mice aged six weeks (18–20 g) were procured from Zhejiang Academy of Medical Sciences (Hangzhou, Zhejiang, China). All mice were housed at 22 ± 2 °C in a SPF environment and were provided standard feed and water ad libitum.

Post-adaptation randomization yielded five groups (*n* = 6 per cage): control check (CK) group, model (DSS) group, meat group, soft sweets group, and jelly group. The CK group and DSS group were fed a normal diet Ain93G (Jiangsu Synergy Pharmaceutical Bioengineering Co., Ltd., Nanjing, China) throughout the whole experiment, while the meat, soft sweets, and jelly groups were fed diets containing 5% (*w*/*w*) CGNPs corresponding to their respective applications. A previous study used 5% carrageenan supplementation in the diet; therefore, this dose was adopted in the present study [4]. From days 7 to 14, 1.5% (*w*/*v*) DSS was added to the drinking water of all groups except the CK group. Disease activity index (DAI) scores were calculated according to Table S1 [22]. On day 13, the feces were collected. Mice were sacrificed on day 14, and colonic contents, cecal contents, and colon tissues were collected and stored at −80 °C for further analysis.

### 2.4. Histopathological Analysis

After Carnoy fixation, the colon samples were embedded in paraffin. Hematoxylin and eosin (H&E) dyestuff was used to dye. The stained sections were examined and imaged using an inverted microscope (Leica DMi1, Leica Microsystems GmbH, Wetzlar, Germany) 4× and 10× magnifications. Histopathological scoring was performed according to the criteria described in Table S2 [23].

### 2.5. ELISA

Fecal or colonic tissue samples were homogenized in PBS according to the instructions, followed by centrifugation for 10 min at 10,000 rpm. The supernatants were collected for ELISA analysis. Lipopolysaccharide (LPS) and lipocalin-2 (LCN2) ELISA kits were purchased from Enzyme-linked (Shanghai, China), and the IL-10 ELISA kit was obtained from ProteinTech (Wuhan, China).

### 2.6. Gut Microbiota Analysis

The colon contents of mice were sent to Novogene (Beijing, China) for 16S rRNA sequencing of the V3–V4 regions. Bioinformatic analysis was performed using QIIME2 on data generated by the Illumina NovaSeq platform (PE300).

### 2.7. SCFA Analysis

SCFAs in the cecal contents of mice were determined using an Agilent 6890N gas chromatograph (Agilent Technologies, Santa Clara, CA, USA) equipped with an HP-INNOWAX column (Agilent Technologies, Santa Clara, CA, USA) [24]. Briefly, cecal contents were homogenized in ultrapure water, followed by centrifugation for 10 min at 10,000 rpm. The supernatants were filtered through a 0.22 μm aqueous-phase membrane filter and subjected to GC analysis. The concentration of SCFAs in the samples was calculated using standard calibration curves including acetate, propionate, butyrate, and valerate.

The analysis was performed using a flame ionization detector (FID) maintained at 300 °C, with the injection port temperature at 250 °C. The airflow and hydrogen flow rates were 400 mL/min and 30 mL/min, respectively. The injection volume was 1 μL.

### 2.8. Statistical Analysis

All graphical representations were generated using GraphPad Prism 8.0. Prior to statistical analysis, data normality was assessed using the Shapiro–Wilk test. One-way analysis of variance (ANOVA) followed by Duncan’s multiple range test was performed using SPSS 26.0 to evaluate the statistical significance of differences among groups (*p* < 0.05). Results are presented as mean ± standard deviation (mean ± SD).

## 3. Results

### 3.1. Structural Characterization of CGNPs for Different Applications

#### 3.1.1. FTIR Spectroscopy

CGNPs from different brands (A, B, C, and D) intended for meat products, soft sweets, and jelly, were sourced from Fujian, Shandong, Guangdong, and Henan, China, respectively.

**Figure 1:**
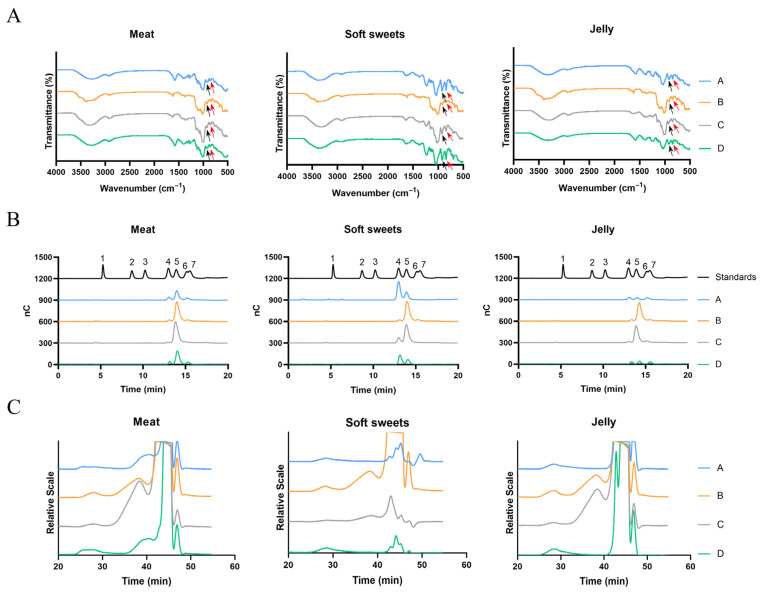
Structural characterization of CGNPs for different applications: (**A**) Infrared spectroscopy; (**B**) monosaccharide composition; (**C**) molecular weight distribution. The black arrows indicate the ether bonds of 3,6-anhydrogalactose, and the red arrows indicate the C-4 sulfate ester groups. Peaks 1–7 correspond to fucose, rhamnose, arabinose, galactose, glucose, mannose, and xylose.

Preliminary identification by FTIR indicated that all twelve samples were kappa-CGN (κ-CGN), as evidenced by the presence of a characteristic absorption peak at 920 cm^−1^ (black arrow in Figure 1A), corresponding to the 3,6-anhydrogalactose ether bond [25]. Additionally, an absorption peak at 850 cm^−1^ (red arrow in Figure 1A), attributed to the C-4 sulfate ester group, further confirmed the presence of κ-CGN [25].

#### 3.1.2. Monosaccharide Composition

The monosaccharide compositions of the twelve CGNPs were further analyzed, and the results are shown in Figure 1B and Table 1. The products from Brands A and D exhibited similar monosaccharide compositions across all three application types. In contrast, all products from Brands B and C contained high levels of glucose. This is likely because these brands use SRC, which contains more cellulose than refined CGN [9]. Cellulose was hydrolyzed to glucose when we analyzed the monosaccharide compositions. Except for Brand B, CGNPs intended for soft sweets contained only galactose and glucose. However, products intended for meat products and jelly from all brands contained not only galactose and glucose, but also mannose. These results suggest that commercial food-grade CGNPs may contain additional components besides CGN itself, possibly including glucomannan, which may facilitate dispersion during processing or improve the gelling performance [9,26].

#### 3.1.3. Molecular Weight

In addition, the molecular weight of CGNPs varied significantly among different brands, but was similar within the same brand (Table 2). Products from Brands A and D exhibited higher molecular weight across all three application types compared to those from Brands B and C, which may be attributed to the presence of more low-molecular-weight substances in the latter (Figure 1C).

#### 3.1.4. One-Dimensional NMR (^1^H NMR) Analysis

Based on the above results, samples from Brands B and C contained substantial amounts of glucose (Figure 1B) and a higher proportion of low-molecular-weight components (Figure 1C). However, the monosaccharide composition of CGN would be expected to consist solely of galactose. Therefore, we aimed to further investigate a sample with a higher galactose content, which would indicate a higher CGN content. Among the remaining samples (A and D), sample A was selected for further NMR analysis.

^1^H NMR results confirmed that CGNPs from Brand A for all three applications were κ-CGN, consistent with the previous FTIR analysis.

**Figure 2:**
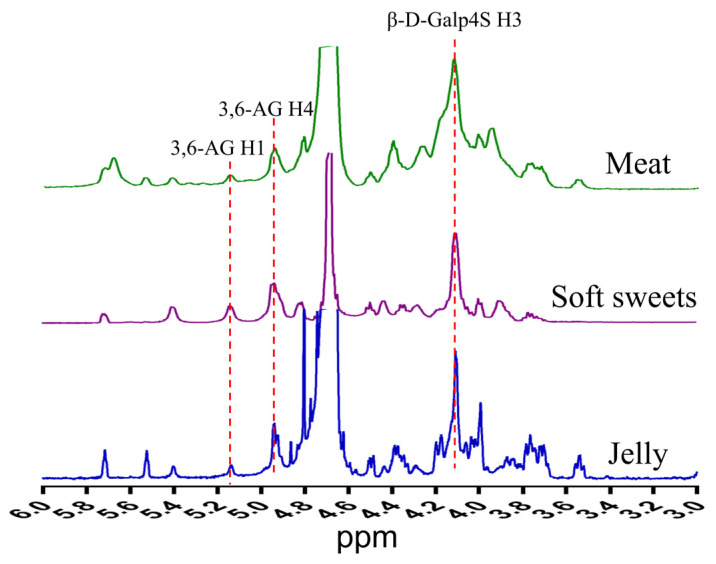
One-dimensional NMR analysis of CGNPs (Brand A) for different applications. ^1^H NMR spectra of meat, soft sweets, and jelly.

At 60 °C, the chemical shift of 3,6-Anhydro-α-D-galactopyranose H-4 (3,6-AG H4), a characteristic residue of κ-CGN, appeared at 5.0 ppm [27]; the signal at 4.1 ppm corresponds to 4-O-Sulfo-β-D-galactopyranose H-3 (β-D-Galp4S H3) (Figure 2).

### 3.2. Effects of CGNPs with Different Applications on DSS-Induced Colitis

**Figure 3:**
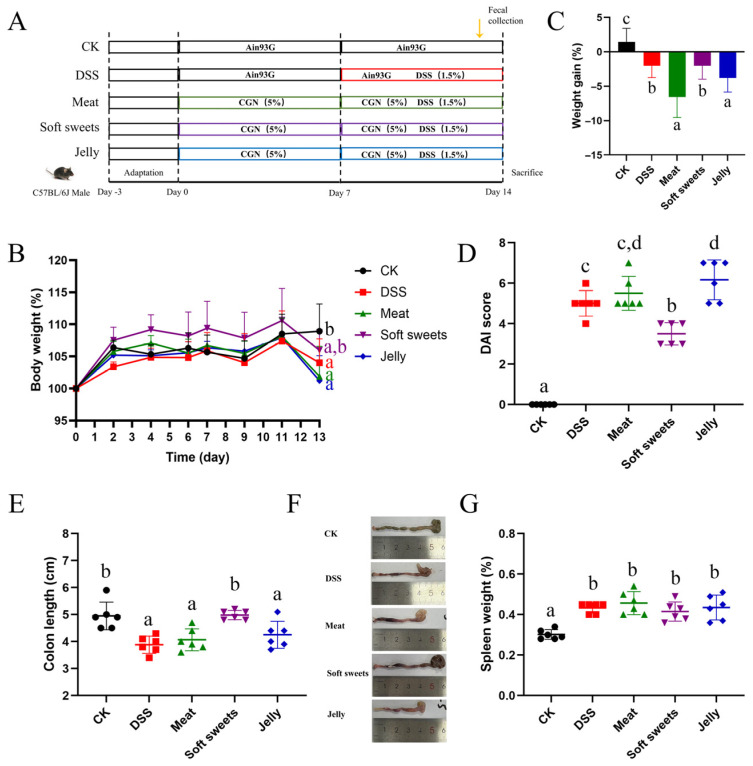
Effects of CGNPs with different applications on DSS-induced colitis: (**A**) Experimental design; (**B**) body weight change relative to initiation; (**C**) body weight change on the final day; (**D**) DAI score; (**E**) colon length; (**F**) images of the colon, recorded from the cecum–colon junction to the distal colon under full extension; (**G**) spleen weight. Different letters indicate significant differences between groups at *p* < 0.05.

While numerous studies report adverse effects of CGN on intestinal health [28,29], data on commercially formulated CGNPs remain limited. Therefore, we investigated the impact of CGNPs on DSS-induced colitis. Samples from Brands A and D were considered for further analysis, as products from Brands B and C contained substantial amounts of glucose. Based on the monosaccharide composition results (Table 1), Brand A, which had a higher galactose content, was ultimately selected for in vivo evaluation. The experimental design is illustrated in Figure 3A.

#### 3.2.1. Body Weight

DSS-induced colitis typically results in body weight loss, diarrhea, bloody stools, and colon shortening [22]. As shown in Figure 3B, the body weight of mice in all groups gradually increased during the acclimation phase, and subsequently declined after DSS administration. By the end of the experimental period, the DSS, meat, and jelly groups showed a significant reduction in body weight compared to the CK group.

#### 3.2.2. DAI Score

The DAI score is commonly used to quantify the severity of DSS-induced colitis. As shown in Figure 3D, all treatment groups exhibited significantly higher DAI scores than the CK group by the end of the experiment. Notably, the soft sweets group showed a significantly lower DAI score compared to the DSS group.

#### 3.2.3. Colon Length

The results showed that colon lengths of the DSS, meat, and jelly groups were significantly shorter than those of the CK group (Figure 3E,F). In contrast, the soft sweets group exhibited a significantly longer colon length than the DSS group, which is consistent with the trends observed in body weight and DAI scores. The protective effect observed in the soft sweets group may be associated with mechanisms mediated by the gut microbiota. On the one hand, CGN is not readily degraded in the upper gastrointestinal tract, and therefore primarily interacts with the gut microbiota in the colon. On the other hand, the monosaccharide composition of the soft sweets group differs from that of the meat and jelly groups. These compositional differences may differentially modulate the structure and function of the gut microbiota, thereby leading to distinct biological effects.

#### 3.2.4. Spleen Index

As the spleen serves as a major hub for immune cells [30], excessive inflammatory responses are often associated with splenomegaly. As shown in Figure 3G, all treatment groups exhibited significantly higher spleen indices compared to the CK group, indicating an enhanced immune response.

#### 3.2.5. Colonic Histopathology

**Figure 4:**
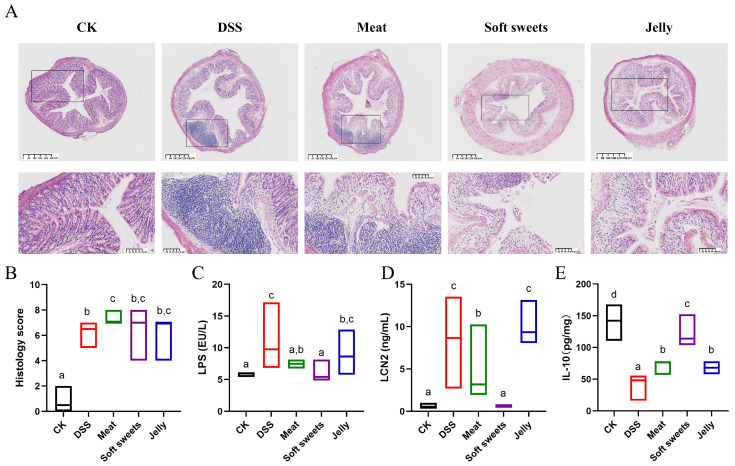
Effects of CGNPs with different applications on histopathology and cytokine levels: (**A**) Representative H&E-stained sections; (**B**) histology score; (**C**) LPS; (**D**) LCN2; (**E**) IL-10. Different letters indicate significant differences between groups at *p* < 0.05.

The results in Figure 4A show that the CK group exhibited intact colonic architecture without inflammatory infiltration. In contrast, all other groups demonstrated significantly increased histopathological scores compared to the CK group (Figure 4B).

#### 3.2.6. Inflammatory Markers

Increased intestinal permeability can lead to the translocation of LPS into intestinal tissues and the systemic circulation [31]. Therefore, LPS level serves as an indicator of both systemic inflammation and intestinal permeability. As shown in Figure 4C, the LPS level in the DSS group was significantly higher than that in the CK group. The meat group exhibited a significantly lower LPS level compared to the DSS group, while no significant difference was observed between the jelly and DSS groups. Notably, the LPS level in the soft sweets group was similar to that in the CK group.

LCN2, a widely recognized inflammatory marker [32], exhibited a trend similar to LPS levels (Figure 4D).

### 3.3. Effects of CGNPs with Different Applications on Gut Microbiota

**Figure 5:**
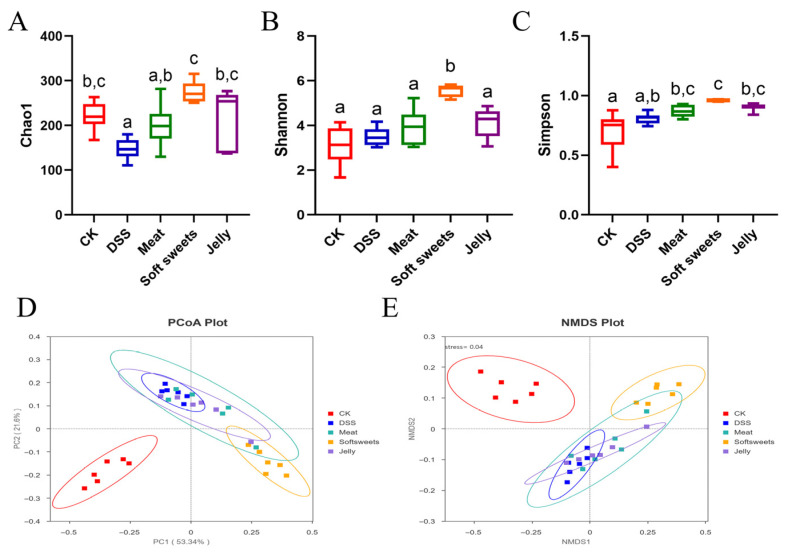
Effects of CGNPs with different applications on gut microbiota diversity: (**A**–**C**) *α* diversity (Chao1, Shannon, and Simpson index); (**D**) PCoA plot; (**E**) NMDS plot. Different letters indicate significant differences between groups at *p* < 0.05.

**Figure 6:**
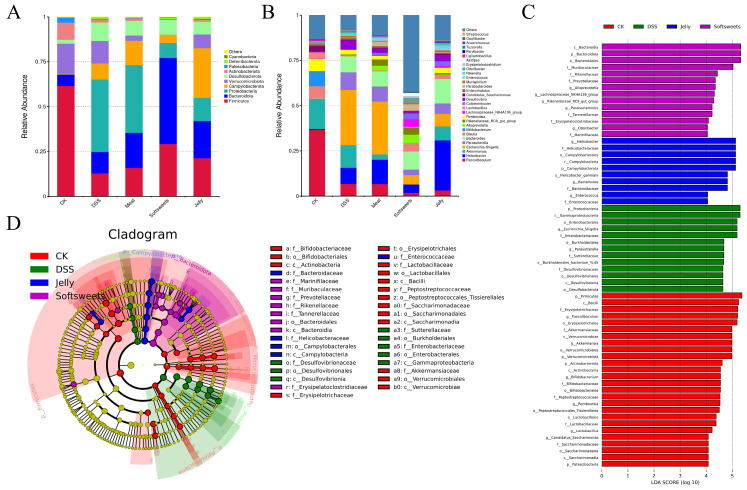
Effects of CGNPs with different applications on gut microbiota: (**A**) relative abundance at the phylum level; (**B**) relative abundance at the genus level; (**C**) LEfSe analysis of differential bacterial taxa among groups, with 63 taxa meeting an LDA score threshold of >4.0; (**D**) taxonomic cladogram.

Gut microbiota dysbiosis is closely associated with ulcerative colitis (UC) [33,34,35,36]. In this study, the gut microbiota composition was analyzed using 16S rRNA gene amplicon sequencing. Alpha diversity represented the microbial richness and diversity. As shown in Figure 5A, the Chao1 index of the DSS group was significantly lower than that of the CK group. However, it was significantly higher in the soft sweets and jelly groups than in the DSS group, suggesting a restorative effect on microbial diversity. Shannon index (Figure 5B) revealed that the soft sweets group exhibited significantly higher diversity than all other groups, which may be attributed to the marked reduction in the abundance of harmful bacteria such as *Escherichia-Shigella* and *Helicobacter* (Figure 6B). Furthermore, except for the DSS group, all other groups showed significantly higher Simpson indexes than the CK group (Figure 5C), suggesting that CGNPs used in different applications may help maintain gut microbial diversity.

Beta diversity reflects the differences in gut microbiota composition. As shown in Figure 5D–E, both PCoA plot (weighted UniFrac distance) and NMDS plot (stress = 0.04) demonstrated a clear separation between DSS group and CK group. Although the soft sweets group was also significantly separated from the DSS group, it did not fully restore the microbiota composition observed in the CK group, and instead retained similarities with the meat and jelly groups.

Figure 6A presents the gut microbiota composition at the phylum level. In the CK group, in addition to the predominant Firmicutes, Bacteroidota, and Actinobacteriota [37], Verrucomicrobiota, which includes the probiotic *Akkermansia muciniphila*, was also present with relatively high abundance. Moreover, Proteobacteria were nearly absent in this group. Firmicutes decreased while Bacteroidota increased after DSS induction, which is consistent with previous studies [38,39]. The reduction in Firmicutes may increase susceptibility to intestinal inflammation [40,41]. Furthermore, DSS and jelly groups were primarily enriched with potentially pathogenic bacteria such as Enterobacteriaceae and Desulfovibrionaceae [42,43], while the soft sweets group selectively promoted polysaccharide-degrading bacteria (Bacteroidota and Prevotellaceae) [44,45] and beneficial bacteria (Muribaculaceae, Lachnospiraceae_NK4A136_group and *Parabacteroides*) [46,47,48] (Figure 6C,D).

### 3.4. Effects of CGNPs with Different Applications on Metabolites

**Figure 7:**
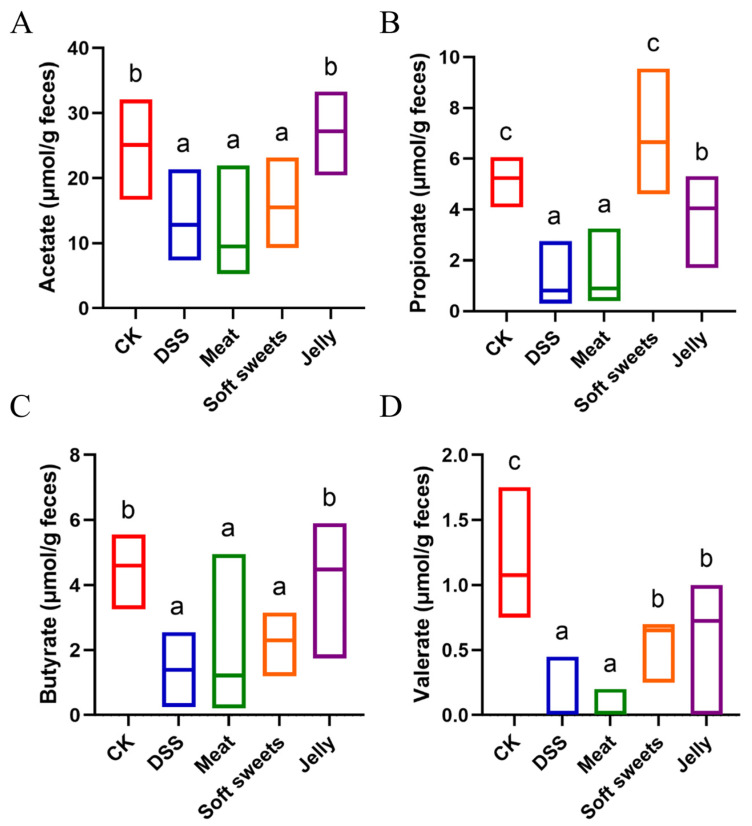
Effects of CGNPs with different applications on SCFAs: (**A**) acetate; (**B**) propionate; (**C**) butyrate; and (**D**) valerate. Different letters indicate significant differences between groups at *p* < 0.05.

**Figure 8:**
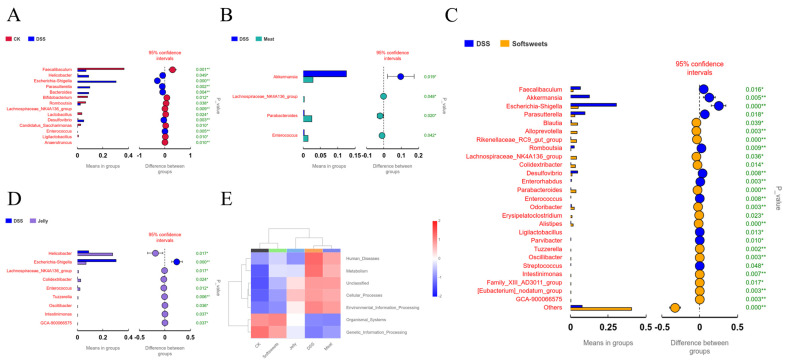
Effects of CGNPs with different applications on gut microbiota composition and predicted functions: (**A**–**D**) relative abundance of microbiota that are significantly altered at the genus level; (**E**) function prediction. *: *p* < 0.05, **: *p* < 0.01.

SCFAs are key metabolites produced by gut microbiota and play a central role in regulating host physiological functions [49]. SCFAs, such as acetate, propionate, and butyrate, not only serve as the primary energy source for colonic epithelial cells but also act as “molecular bridges” linking the microbiota and host health by modulating immune responses, maintaining intestinal barrier integrity, and influencing systemic metabolic homeostasis [50]. As shown in Figure 7A–D, SCFA levels in the DSS group were significantly lower than those in the CK group, which may be attributed to the marked reduction in SCFA-producing bacteria in the DSS group (Figure 8A), including *Bifidobacterium*, Lachnospiraceae_NK4A136_group, *Lactobacillus*, and *Ligilactobacillus* [47,51,52,53]. Meanwhile, *Desulfovibrio* was significantly enriched in the DSS group (Figure 8A); this bacterium produces hydrogen sulfide, which inhibits SCFA production [54]. The meat group contributed minimally to SCFA production, consistent with its gut microbiota composition being most similar to that of the DSS group (Figure 6B and Figure 8B). The soft sweets group primarily contributed propionate, which is associated with enrichment in *Parabacteroides*, *Blautia*, and *Alloprevotella* (Figure 8C). The former two can directly produce propionate [55,56], while *Alloprevotella* generates succinate that can be further converted into propionate [57]. In contrast, the jelly group mainly contributed acetate and butyrate, which can be attributed to the increased abundance of Lachnospiraceae_NK4A136_group and *Oscillibacter* (Figure 8D) [58,59].

In addition, the CK and soft sweets groups exhibited significant enrichment in Organismal Systems and Genetic Information Processing pathways (Figure 8E). The enrichment of Organismal Systems pathway suggests that normal microbiota actively participate in host physiological regulation, such as maintaining immune homeostasis and promoting the synthesis of neurotransmitters [60]. The Genetic Information Processing category encompasses DNA replication, repair, transcription, and translation [61]. The enrichment of these pathways in the CK group indicates higher genomic stability of the gut microbiota, allowing for more effective responses to external stressors and maintenance of genetic integrity. In contrast, the reduction in these pathways in the DSS group may imply that the microbial community is under stress, with increased DNA damage and insufficient repair capacity, potentially leading to the accumulation of mutations or the horizontal transfer of virulence genes, which could further disrupt microbial structure and function. The DSS and meat groups were mainly enriched in Human Diseases, Metabolism, and Environmental Information Processing pathways. The Human Diseases pathway enrichment may be associated with the upregulation of IBD-related genes. The Metabolism pathway enrichment may reflect alterations in microbial metabolic activity, such as reduced SCFAs (Figure 7A–D) or dysregulated bile acid metabolism, both of which can affect host metabolism. The Environmental Information Processing pathway may involve activation of signaling pathways such as NF-κB, thereby promoting the release of pro-inflammatory cytokines (Figure 4D).

## 4. Discussion

CGN’s functional properties are primarily determined by its molecular structure and degree of sulfation. The predominant types of CGN in food applications are κ, ι, and λ forms. κ-CGN exhibits high gel strength and is widely used in meat products. Its linear molecular chain and 1,3-linked 3,6-anhydrogalactose units form a rigid gel network that enhances the rheological properties and water-holding capacity of meat products. ι-CGN forms elastic gels and is primarily used in jelly products. Its sulfate groups at the C2 and C4 positions contribute to the flexibility of the gel [8]. Moreover, λ-CGN, typically used as a thickening agent in dairy products [16], relies on its high degree of sulfation to achieve electrostatic stabilization.

The combination of different additives with CGN may influence the way these products interact with the gut microbiota [62,63], thereby potentially affecting SCFA production and the severity of colitis. Based on the monosaccharide compositions of CGNPs intended for meat products and jelly (Table 1), together with reported monosaccharide profiles of hydrocolloids commonly co-applied with CGN, CGNPs intended for jelly may contain konjac gum, whereas CGNPs intended for meat products may contain a higher proportion of SRC, which is richer in cellulose. However, these compositional inferences were not structurally confirmed in the present study. Previous studies have shown that konjac gum may increase the abundance of *Lachnoclostridium* [64] and is associated with enhanced butyrate production [65], which is partly consistent with the SCFA profile observed here. Nevertheless, such changes were not accompanied by an alleviation of colitis in the present study. By contrast, CGNPs intended for soft sweets did not exacerbate colitis in mice and were associated with enrichment in *Parabacteroides*, *Blautia*, and *Alloprevotella* (Figure 8C). These taxa have been reported to be involved in propionate-related metabolic pathways, which is consistent with the propionate profile observed in this study, although the underlying mechanisms require further investigation.

This study also has several limitations. First, only male mice were used in this study, and potential sex-dependent responses were not evaluated. Second, although SCFAs were analyzed, other important microbial metabolites such as bile acids were not investigated. Third, immune-cell profiling (e.g., Th17 or Treg cells) was not performed, which limits mechanistic interpretation of the host immune responses. Finally, detailed compositions of proprietary CGNP products used in this study were not fully disclosed, which may affect the interpretation of the observed differences among applications. Future studies incorporating multi-omics approaches and more comprehensive immune analyses are needed to further elucidate the underlying mechanisms.

## 5. Conclusions

The biological effects of CGNP are closely related to its structural characteristics. The κ-type backbone, combined with sulfate groups, provides the basic gelling properties, while monosaccharide modifications significantly alter its functional attributes. The effects of CGNPs on colitis vary depending on their intended application. CGNPs intended for soft sweets mitigated DSS-induced Firmicutes/Bacteroidota imbalance and promoted propionate-producing bacteria. CGNPs intended for jelly enhanced intestinal energy metabolism but did not alleviate colitis, whereas CGNPs intended for meat products exacerbated metabolic disturbances and inflammation. These findings may have implications for the selection and formulation of CGNP-containing food products in the food industry. However, because the present study was performed in a DSS-induced mouse model, the results should not be directly extrapolated to humans. Further studies are needed to identify the specific structural features or metabolites underlying these effects and to assess their relevance in humans.

## Figures and Tables

**Table 1:** Quantitative analysis of monosaccharides of CGNPs with different applications.

Monosaccharide (mol %)		Meat	Soft Sweets	Jelly
	Gal	16.42 ± 0.84	66.51 ± 1.57	27.04 ± 2.56
A	Glc	57.97 ± 2.85	33.49 ± 1.57	27.10 ± 2.51
	Man	25.62 ± 3.69	-	45.86 ± 5.07
	Gal	5.08 ± 0.12	4.57 ± 0.13	4.54 ± 0.12
B	Glc	81.72 ± 1.77	81.40 ± 2.28	82.68 ± 2.07
	Man	13.20 ± 1.89	14.04 ± 2.41	12.78 ± 2.19
	Gal	2.38 ± 0.06	16.65 ± 0.20	4.23 ± 0.18
C	Glc	86.90 ± 1.93	83.35 ± 0.20	79.32 ± 3.12
	Man	10.72 ± 1.99	-	16.45 ± 3.30
	Gal	11.88 ± 0.49	59.56 ± 1.59	24.22 ± 2.57
D	Glc	66.41 ± 2.62	40.44 ± 1.59	35.65 ± 3.73
	Man	21.71 ± 3.11	-	40.13 ± 6.30

A, B, C, and D represent different brands.

**Table 2:** Molecular weight of CGNPs with different applications.

Molecular Weight (kDa)	Meat	Soft Sweets	Jelly
A	532.9 (±1.170%)	655.3 (±3.535%)	594.6 (±0.582%)
B	223.9 (±0.602%)	201.8 (±0.470%)	329.9 (±0.663%)
C	132.6 (±0.523%)	142.4 (±1.068%)	186.7 (±0.381%)
D	761.6 (±1.119%)	577.5 (±5.112%)	652.6 (±0.649%)

A, B, C, and D represent different brands.

## Data Availability

The original contributions presented in the study are included in the article/Supplementary Materials, further inquiries can be directed to the corresponding author.

## Supplementary Materials

The following supporting information can be downloaded at: https://www.mdpi.com/article/10.3390/foods15071131/s1. Table S1: The disease activity index (DAI) scores; Table S2: The histological scores; Table S3: Alpha-diversity values.

## Abbreviations

CGN: carrageenan; CGNPs: carrageenan preparations; DAI: disease activity index; LBG: locust bean gum; KG: konjac gum; XG: xanthan gum; SRC: semi-refined CGN; CMC: carboxymethyl cellulose; GG: guar gum; SA: sodium alginate; GA: gum arabic; IBD: inflammatory bowel diseases; SCFAs: short-chain fatty acids; DSS: dextran sulfate sodium.
