# Supplementary material for: Differential Effects of Commercial Food-Grade Carrageenan Preparations on DSS-Induced Colitis and Gut Microbiota in Mice
Source: Foods. 2026 Mar 25;15(7):1131. doi: 10.3390/foods15071131 (PMC13074121; doi:10.3390/foods15071131)
Supplement: Supplementary file 1 [file foods-15-01131-s001.zip › foods-4190379-supplementary.pdf]

Supplementary Material

Table S1 The disease activity index (DAI) scores.

Score	Weight loss (%)	Stool consistency	Blood in feces
**0**	0	Normal	No bleeding
**1**	0.1~5.0	Soft but still formed	Slight bleeding
**2**	5.0~10.0	Very soft; wet	Excessive bleeding
**3**	>10.0	Watery diarrhea	Gross bleeding

DAI = (Weight loss scores + Stool consistency scores + Blood in feces scores) / 3

Table S2 The histological scores.

Score	Epithelial cell	Inflammatory infitration
**0**	None	Normal
**1**	Loss of goblet cells in small area	Infiltrate around crypt basis
**2**	Loss of goblet cells in large area	Infiltrate to mucosa
**3**	Loss of crypts in small area	Extensively infiltrate to mucosa
**4**	Loss of crypts in large area	Infiltrate to submucosa

Histological scores = Epithelial cell scores + Inflammatory infiltration scores

Table S3 Alpha-diversity values.

Group	Sample ID	Chao1	Shannon	Simpson
CK	CK-1	263	4.146	0.773
	CK-2	216.913	3.044	0.876
	CK-3	215.053	3.219	0.756
	CK-4	167	2.738	0.649
	CK-5	221.636	1.68	0.401
	CK-6	242.667	3.77	0.751
DSS	DSS-1	149	3.718	0.815
	DSS-2	163.048	4.171	0.878
	DSS-3	180	3.16	0.774
	DSS-4	138.067	3.018	0.817
	DSS-5	144.615	3.585	0.816
	DSS-6	110.75	3.295	0.744
Meat	Meat-1	281	5.222	0.929
	Meat-2	130.273	3.031	0.802
	Meat-3	194.4	3.158	0.887
	Meat-4	207.545	4.232	0.921
	Meat-5	202.714	4.045	0.844
	Meat-6	183.565	3.827	0.829
Soft sweets	Soft sweets-1	254.5	5.684	0.949

	Soft sweets-2	250.667	5.318	0.958
	Soft sweets-3	286.452	5.154	0.966
	Soft sweets-4	315.4	5.636	0.97
	Soft sweets-5	283.545	5.766	0.957
	Soft sweets-6	257.833	5.832	0.963
Jelly	Jelly-1	265.625	4.866	0.917
	Jelly-2	137.125	4.275	0.838
	Jelly-3	276.714	3.676	0.91
	Jelly-4	138.056	4.54	0.908
	Jelly-5	262.867	3.065	0.935
	Jelly-6	245.143	4.288	0.917
